# *Streptococcus pneumoniae* and *Haemophilus influenzae* in paediatric meningitis patients at Goroka General Hospital, Papua New Guinea: serotype distribution and antimicrobial susceptibility in the pre-vaccine era

**DOI:** 10.1186/s12879-015-1197-0

**Published:** 2015-10-27

**Authors:** Andrew R. Greenhill, Suparat Phuanukoonnon, Audrey Michael, Mition Yoannes, Tilda Orami, Helen Smith, Denise Murphy, Christopher Blyth, John Reeder, Peter Siba, William Pomat, Deborah Lehmann

**Affiliations:** Papua New Guinea Institute of Medical Research, Goroka, Papua New Guinea; Telethon Kids Institute, The University of Western Australia, Perth, Australia; School of Applied and Biomedical Sciences, Federation University, Churchill, Australia; The Walter and Eliza Hall Institute of Medical Research, Melbourne, Australia; Queensland Department of Health, Forensic and Scientific Services, Coopers Plains, Australia; School of Paediatrics and Child Health, The University of Western Australia, Perth, Australia; Princess Margaret Hospital for Children, Perth, Australia; PathWest Laboratory Medicine, Perth, Western Australia Australia; Department of Epidemiology and Preventative Medicine, Monash University, Melbourne, Australia

**Keywords:** Meningitis, Low-income, Pneumococcal conjugate vaccine

## Abstract

**Background:**

Bacterial meningitis remains an important infection globally, with the greatest burden in children in low-income settings, including Papua New Guinea (PNG). We present serotype, antimicrobial susceptibility and outcome data from paediatric meningitis patients prior to introduction of *Haemophilus influenzae* type b (Hib) and pneumococcal conjugate vaccines (PCVs) in PNG, providing a baseline for evaluation of immunisation programs.

**Methods:**

Cerebrospinal fluid (CSF) was collected from children admitted to Goroka General Hospital with suspected meningitis between 1996 and 2005. Culture and sensitivity was conducted, and pneumococci and *H. influenzae* were serotyped. Laboratory findings were linked to clinical outcomes.

**Results:**

We enrolled 1884 children. A recognised pathogen was identified in 375 children (19.9 %). *Streptococcus pneumoniae* (*n* = 180) and Hib (*n* = 153) accounted for 88.8 % of pathogens isolated. 24 different pneumococcal serogroups were identified; non-PCV types 2, 24 and 46 accounted for 31.6 % of pneumococcal meningitis. 10- and 13-valent PCVs would cover 44.1 % and 45.4 % of pneumococcal meningitis respectively. Pneumococcal isolates were commonly resistant to penicillin (21.5 %) and 23 % of Hib isolates were simultaneously resistant to ampicillin, co-trimoxazole and chloramphenicol. The case fatality rate in patients with a recognised bacterial pathogen was 13.4 % compared to 8.5 % in culture-negative patients.

**Conclusions:**

If implemented in routine expanded programme of immunisation (EPI) with high coverage, current PCVs could prevent almost half of pneumococcal meningitis cases. Given the diversity of circulating serotypes in PNG serotype replacement is of concern. Ongoing surveillance is imperative to monitor the impact of vaccines. In the longer term vaccines providing broader protection against pneumococcal meningitis will be needed.

**Electronic supplementary material:**

The online version of this article (doi:10.1186/s12879-015-1197-0) contains supplementary material, which is available to authorized users.

## Background

Bacterial meningitis is an important cause of morbidity and mortality in children in low-income countries [1]. The two most important etiological agents of bacterial meningitis are *Streptococcus pneumoniae* and *Haemophilus influenzae* type b (Hib). In total, *S. pneumoniae* kills over 800,000 children (<5 years old) each year, with meningitis being the most common severe form of invasive pneumococcal disease (IPD) [2]. Prior to introduction of Hib conjugate vaccine there were ~2.2 million cases of serious Hib disease annually, the vast majority in low-income settings [3]. In low-income countries the case fatality rate (CFR) for acute bacterial meningitis is commonly above 30 % and usually higher for pneumococcal than Hib meningitis [2, 4–7]. In children who survive acute bacterial meningitis, neurological complications are common with approximately one in four children in developing countries suffering long-term neurological sequelae following pneumococcal meningitis [8].

Vaccines have been developed to reduce mortality and morbidity due to IPD and Hib disease. Hib vaccine is effective in high and low socio-economic settings. In The Gambia the incidence of Hib meningitis remains below 5 cases/100,000 14 years after the introduction of a three dose course of Hib vaccine [9, 10]. In Indigenous children in Western Australia the introduction of Hib vaccine in 1993 resulted in a significant decline in hospital admission for meningitis [11]. Despite the availability of conjugate Hib vaccines since the 1980s, there has been a delay in their inclusion in national immunisation programs in many low-income countries [12].

The 23-valent pneumococcal polysaccharide vaccine (PPV) (serotypes 1, 2, 3, 4, 5, 6B, 7 F, 8, 9 N, 9 V, 10, 11, 12 F, 14, 15B, 17 F, 18C, 19 F, 19A, 20, 22 F, 23 and 33 F) is generally considered to be poorly immunogenic in children under 2 years old. The pneumococcal conjugate vaccines (PCVs) consist of serotype-specific polysaccharides conjugated to a protein to improve immunogenicity in children <2 years old. Introduction of the heptavalent conjugate vaccine, PCV7 (serotypes 4, 6B, 9 V, 14, 18C, 19 F and 23 F) into national immunisation programs has reduced the incidence of IPD in a number of industrialised countries [13, 14]. Studies have demonstrated efficacy of a 9-valent PCV (PCV7 serotypes plus serotypes 1 and 5) in two African settings [15, 16]. PCV7 has been superseded in recent years by PCV10 (PCV7 serotypes plus 1, 5 and 7 F) and PCV13 (PCV10 serotypes plus 3, 6A, and 19A). Rollout of these higher valency vaccines is now occurring in low-income countries.

Previous studies in PNG have shown *S. pneumoniae* and Hib to be the most common causes of bacterial meningitis and have provided data on serotype distribution and antimicrobial susceptibility of isolates [6, 17]. With the introduction of Hib vaccine into the PNG national program in 2008 and rollout of PCV13 commencing in 2014, ongoing surveillance of serotype distribution and antimicrobial susceptibility is essential to ensure optimal prevention and treatment strategies.

Acute flaccid paralysis surveillance has been conducted in Goroka in the PNG highlands since 1996 as part of the global polio eradication campaign. As such, suspected cases of meningitis have been investigated, providing data of Hib and pneumococcal meningitis, serotype distribution and antimicrobial susceptibility in the pre-vaccine era. We report here on data collected from children admitted to Goroka General Hospital (GGH; now called Eastern Highlands Provincial Hospital) between August 21^st^, 1996 and June 17^th^, 2005.

## Methods

### Setting and study population

GGH is the referral hospital for Eastern Highlands Province (population ~433,000 in 2000) of PNG. The provincial capital Goroka (altitude 1546 m asl) has a population of ~20,000 (70,000 in the surrounding district). The majority of people are subsistence farmers, with the major cash crop in the province being coffee.

We have conducted surveillance of suspected meningitis in children aged <15 years admitted to GGH since 1996. Case identification was based on any of the following clinical signs or symptoms: history of convulsion, altered level of consciousness, neck stiffness, bulging or tense fontanelle at rest, focal neurological signs associated with history of recent febrile illness, refusal or inability to feed associated with a febrile illness, or paediatrician’s suspicion of meningitis in the absence of above signs and symptoms. We documented whether patients were discharged, absconded or died from the hospital records.

### Laboratory methods

Cerebrospinal fluid (CSF) was collected via lumbar puncture using aseptic technique. Where possible CSF was collected prior to administration of antibiotics in the hospital. Samples were processed at the PNG Institute of Medical Research (PNGIMR) as soon as possible after collection. Standard methods, namely microscopy and bacterial culture were used to diagnose meningitis and determine etiological agents: these methods are well established in this setting [17]. Microscopy included cell counts (polymorphonuclear neutrophils, lymphocytes and erythrocytes) and Gram stain of pelleted CSF. Evidence of prior antimicrobial treatment was garnered through an assay in which a disk impregnated with the patient’s CSF was placed on an agar plate seeded with *S. aureus* ATCC 25923. *H. influenzae* was serotyped at PNGIMR using *H. influenzae* antisera a-f (Remel, Thermo Fisher Scientific, Australia)*.* Pneumococci were serogrouped at PNGIMR by the Quellung reaction (Statens Serum Institut, Copenhagen, Denmark) and a subset sent to Queensland Health Pathology Service (Brisbane, Australia) for confirmation and factor typing.

Antibiotic susceptibility testing was conducted by disk diffusion (Oxoid, Thermo Fisher Scientific, Australia) following CLSI guidelines [18]. Isolates were tested for susceptibility to chloramphenicol, tetracycline, co-trimoxazole, ceftriaxone, ampicillin (*H. influenzae* only), oxacillin (*S. pneumoniae* only) and erythromycin (*S. pneumoniae* only). Minimum inhibitory concentration (MIC) testing was conducted using E-test (AB Biodisk, Sweden) following CLSI guidelines [18]. MIC tests were conducted on pneumococcal isolates to determine susceptibility to penicillin, chloramphenicol and cotrimoxazole, with a subset tested for susceptibility to tetracycline, ceftriaxone and erythromycin. *H. influenzae* isolates had MICs determined for ampicillin, chloramphenicol and cotrimoxazole. At the time this study was conducted (1996–2005) the MIC for resistance to penicillin in *S. pneumoniae* was ≥2 μg/ml (as it remains currently for non-meningitis cases). CSF pneumococcal isolates now are considered resistant to penicillin at MIC ≥0.12 μg/ml [18]. We applied the current guidelines when determining resistance to penicillin in pneumococcal isolates. Serotyping and sensitivity testing was conducted at the time of isolation.

Data were double-entered using FoxPro 8 (Microsoft Corp, USA) and analysed using Excel (Microsoft Corp, USA). The Yates chi-square test was used to compare proportions between groups of interest. Ethics approval was granted by the PNG Medical Research Advisory Committee to conduct CSF bacterial culture and biochemistry as part of the acute flaccid paralysis surveillance. The need for written informed consent was waivered by the ethics committee as this work was conducted as part of good clinical care of the patients.

## Results

Between August 1996 and June 2005 CSFs were collected from 1884 patients: 1126 males and 758 females. The median age of patients was 6 months (lower quartile 3 months, upper quartile 12 months). Bacteria were isolated from 480 (25.5 %) samples; two organisms were isolated from five CSF samples, resulting in 485 bacterial isolates. *S. pneumoniae* (180) and *H. influenzae* (165, of which 153 were Hib) were the most commonly isolated pathogens, accounting for 91.5 % of recognised pathogens isolated. Other bacterial pathogens included Enterobacteriaceae (14), β-haemolytic streptococci (8) and *Acinetobacter* spp (6) (Table 1). Thirty-two isolates were considered probable contaminants: 12 *Staphylococcus epidermidis,* 10 *Bacillus* spp., 4 non-*aeruginosa* pseudomonads, 1 *Micrococcus* sp. and 5 non-specified. A further 76 isolates were considered possible pathogens (68 *Staphylococcus aureus*, 5 viridans streptococci, 2 *Enterococcus faecalis* and 1 *Klebsiella oxytoca*). The temporal distribution of *S. aureus* isolates was indicative of contamination for a large proportion of these isolations: 45 of 68 isolations occurred in 2004 and 2005 and 94.4 % (34 of 36 that had cell count conducted within this period) occurred in the absence of polymorphonucleocytes (PMN) in the CSF. In total, 375 CSF (19.9 %) samples were positive for a recognised bacterial pathogen (excluding probable contaminants and possible pathogens). In two of these samples multiple bacteria were isolated, resulting in a total of 377 recognised pathogens, as listed in Table 1.

**Table 1:** Pathogens isolated from CSF of children admitted to Goroka General Hospital with meningitis. A total of 377 pathogens were isolated, with an additional 108 isolates considered contaminants (not shown in table)

Pathogen	Number
*Streptococcus pneumoniae*	180
*Haemophilus influenzae*	165
(Hia 9; Hib 153; Hic 2; NT 1)	
*Neisseria meningitidis*	1
Group A *Streptococcus*	6
Group B *Streptococcus*	2
*Escherichia coli*	4
*Klebsiella pneumoniae*	3
*Enterobacter cloacae*	1
*Proteus* sp.	1
*Proteus mirabilis*	1
*Salmonella* sp.	2
*Providencia* sp.^a^	1
*Citrobacter* sp.^a^	1
*Pseudomonas aeruginosa*	2
*Acinetobacter calcoaceticus*	2
*Acinetobacter lwoffii*	4
Non-haemolytic *Streptococcus* ^b^	1

Hia, Hib and Hic correspond to the serotype of *H. influenzae*

*NT* non-typable

^a^Isolated from the same CSF sample

^b^Coinfection with *E. coli*

Of the 1404 culture negative CSF samples, in 263 samples PMN were >10 × 10^6^/l (10–100 × 10^6^/l in 147 samples; >100 × 10^6^/l in 116 samples). We also detected antimicrobial activity in the CSF of 126 of 1775 samples tested (including 10 samples with high PMN counts). A summary of microscopy results is provided in Additional file 1: Table S1.

Outcomes (died, discharged or absconded) were documented for 1351 patients (71.7 % of participants). 71 (5 %) children were taken home while still sick. The CFR during hospitalisation among remaining children was 9.5 %. The CFR for patients in whom a recognised bacterial pathogen was isolated (i.e. excluding possible pathogens and probable contaminants) was 13.4 % compared to a CFR of 8.5 % in patients with no bacterial pathogen isolated (probable contaminant or no bacteria isolated) (*χ*^2^ = 4.94, degrees of freedom (df) = 1, *p* = 0.026). The CFR for pneumococcal meningitis was 15.4 %, and 8.9 % for patients with Hib meningitis (*χ*^2^ = 1.82, df = 1, *p* = 0.177).

Significant differences in age distribution of pneumococcal and Hib meningitis were noticed (Table 2). Pneumococcal meningitis most frequently occurred in infants aged < 6 months (52.8 % of all pneumococcal meningitis compared with 36.2 % of Hib meningitis, *χ*^2^ = 8.50, 1df, *p* = 0.004) whereas Hib meningitis was most frequent in children aged 6–11 months (50.0 % of Hib cases compared with 20.0 % of pneumococcal meningitis cases, *χ*^2^ = 31.85, 1df, *p* = 0.000).

**Table 2:** Number of samples, male:female ratio, number (isolation rate %) of *S. pneumoniae* and *H. influenzae*, total number of deaths, discharges and case fatality rate (CFR) for different age groups. Pneumococcal serotypes that were isolated more than once in an age group are listed

Age (mo)	Number	M:F	Pnc (%)	Hib (%)	Predominant Pnc serogroups (n)	Died	Discharged	CFR
0–5	892	1.60:1	95 (10.7)	55 (6.2)	2 (16); 5 (15); 14 (5); 12 (5); 7(4); 9(4)	54	519	9.4
6–11	452	1.40:1	36 (8.0)	76 (16.8)	2 (5); 24 (5); 46 (4); 7 (3)	24	297	7.5
12–23	187	1.25:1	17 (9.1)	12 (6.4)	6 (4); 2 (2)	18	114	13.6
24–59	197	1.37:1	18 (9.1)	5 (2.5)	18(2)	17	128	11.7
60+	145	1.56:1	14 (9.0)	4 (2.7)	2 (2); 7 (2); 18 (2)	9	98	8.4
ANR	11	2.67:1	0 (0)	1 (9.1)		0	2	0.0

CFR based on available data in hospital records. Denominator excludes those who absconded

*ANR* Age not recorded. Pnc: pneumococcus

### Serotype distribution of S. pneumoniae and H. influenzae

Of the 180 pneumococcal isolates, 171 were serogrouped; yielding 24 different serogroups (29 serotypes) and one non-typable isolate (Additional file 2: Table S2). The most common serogroups were 2 (17.5 % of all serogrouped isolates), 5 (10.5 %), 46 (8.8 %) and 7 (7.0 %).

**Fig. 1:**
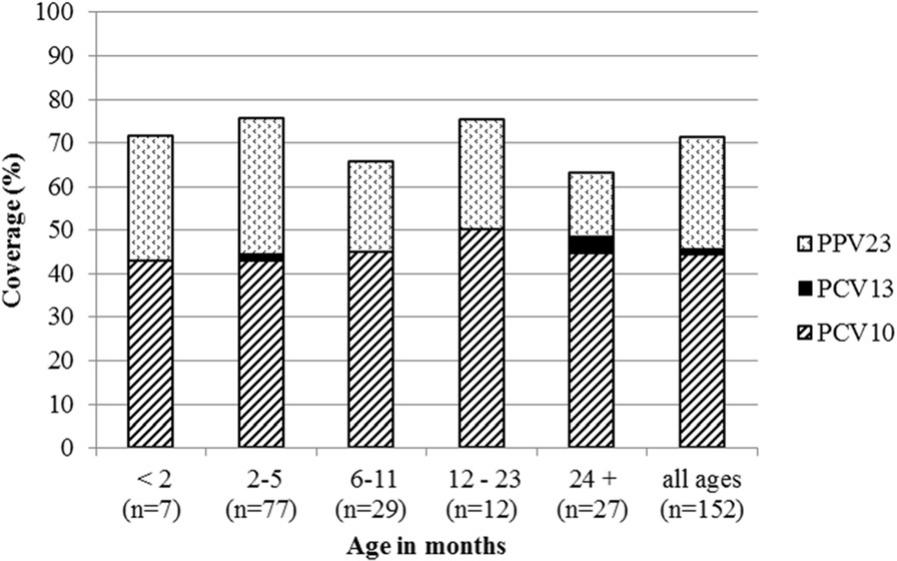
The proportion of cases with culture-confirmed pneumococcal meningitis that would be covered by 10vPCV, by the additional coverage offered by PCV13 and by PPV by age group. The number in parenthesis (n) represents the number of cases in that age group for which serogroup/type data are available. In three age groups (<2, 6–11, and 12–23 months) PCV13 would offer no additional protection above that of PCV10. The serotype 6A was not tested with 6C antisera, thus reported as 6A in this analysis

The proportion of cases of culture-confirmed pneumococcal meningitis that would be covered by 10- and 13-valent PCVs and PPV is shown in Fig. 1. Where factor type was relevant, non-factor-typed isolates were excluded from analysis, resulting in 152 cases. The PCV10 would cover 44.1 % of all cases in our setting in PNG; an additional 1.3 % of cases (45.4 %) would be covered by PCV13.

Hib accounted for 153 (93 %) of the 165 of *H. influenzae* isolated from CSF; nine isolates (5.5 %) were serotype a, two (1.2 %) serotype c and one (0.6 %) non-typable.

### Antimicrobial susceptibility

Susceptibility testing was conducted by disk diffusion on 177 *S. pneumoniae* isolates. Reduced susceptibility to oxacillin was observed in 34 (17.2 %) *S. pneumoniae* isolates; resistance to other antibiotics was uncommon. Pneumococcal resistance to penicillin (MIC determined by E-test) was observed in 21.5 % of isolates (Table 3). Some serotypes commonly exhibited reduced susceptibility to penicillin: serogroups/types 19 (5/6 isolates), 6 (9/11), 10 (3/4), 14 (5/8) 24 (4/9) and 9 (2/5). The one pneumococcal isolate with reduced susceptibility to ceftriaxone (serotype 14) was also resistant to penicillin and cotrimoxazole; three other isolates were resistant to both penicillin and cotrimoxazole. All four tetracycline-resistant *S. pneumoniae* were also resistant to cotrimoxazole; two isolates were resistant to additional antibiotics (one to penicillin and one to chloramphenicol).

**Table 3:** Proportion of *S. pneumoniae* and *H. influenzae* isolates with reduced susceptibility to antibiotics, determined by minimum inhibitory concentration (MIC)

	Number tested	Intermediate resistant	Resistant	Median MIC RS isolates
*S. pneumoniae*	(*n* = 180)			
Penicillin	177	NA	38 (21.5 %)	0.25
Cotrimoxazole	176	8 (4.5 %)	7 (4.0 %)	2/38
Tetracycline	96	0 (0 %)	4 (4.2 %)	24
Chloramphenicol	176	NA	4 (2.3 %)	16
Ceftriaxone	124	1 (0.8 %)	0 (0 %)	1
Erythromycin	94	0 (0 %)	0 (0 %)	NA
*H. influenzae*	(*n* = 165)			
Ampicillin	162	8 (4.9 %)	46 (28.4 %)	4
Chloramphenicol	162	NA	51 (31.5 %)	16
Cotrimoxazole	162	8 (4.9 %)	55 (34.0 %)	>32/608

CLSI breakpoints (μg/ml) for resistance (R) and intermediate resistance (IR) [18]

*S. pneumoniae*: penicillin R ≥0.12; cotrimoxazole IR =1/19–2/38, R ≥4/76; tetracycline IR =4, R ≥8; chloramphenicol R ≥8; ceftriaxone IR =1, R ≥2; erythromycin IR =0.5, R ≥1

*H. influenzae*: ampicillin IR =2, R ≥4; chloramphenicol IR =4, R ≥8; cotrimoxazole; IR =1/19–2/38, R ≥4/76

*RS* reduced susceptibility (intermediate resistance and resistance)

All 165 *H. influenzae* isolates had β-lactamase test conducted, and susceptibility testing was conducted by disk diffusion on 163 isolates. Fifty-three (32.5 %) isolates were β-lactamase positive. All resistant *H. influenzae* isolates were Hib. All β-lactamase positive isolates were resistant to ampicillin and cotrimoxazole by disk diffusion, 52/53 were also resistant to chloramphenicol and 51/52 resistant to tetracycline. One-hundred and sixty-two isolates had MICs determined for ampicillin, chloramphenicol and cotrimoxazole. One-third of isolates demonstrated reduced susceptibility to ampicillin, with a similar proportion of isolates having reduced susceptibility to cotrimoxazole and chloramphenicol (Table 3). Multiple resistance was common in *H. influenzae* isolates, with 38 (23 %) isolates resistant to ampicillin, chloramphenicol and cotrimoxazole. An additional 15 isolates showed multiple resistance or intermediate resistance to two or three of those antibiotics.

Only disk diffusion testing was conducted for tetracycline (*n* = 162) and ceftriaxone (*n* = 74). Fifty-seven *H. influenzae* isolates were resistant to tetracycline (35 %) with one additional isolate demonstrating intermediate resistance. Fifty-two of the tetracycline-resistant isolates were also resistant to ampicillin, chloramphenicol and cotrimoxazole. Four isolates (5 %) were non-susceptible to ceftriaxone.

Analysis was conducted to determine if the rate of antibiotic resistance increased during the study period, using the end of 2000 as the approximate mid-point of the surveillance (Table 4). There was no statistical difference in the proportion of pneumococci that were resistant pre-2001 compared to the later period (2001–June 2005). However, the proportion of *H. influenzae* that were resistant to ampicillin increased significantly between the two periods (Table 4).

**Table 4:** Proportion of *S. pneumoniae* and *H. influenzae* (%) with reduced antimicrobial susceptibility. Bacteria isolated in first half of study (1996–2000) were compared to those from the second half of study (2001–2005). Yates chi-square test was used to compare the proportion of strains that were resistant in the first half of the study period (1996–2000) with that in the second half of the study period (2001–2005)

	1996–2000	2001–2005	*P* value
*S. pneumoniae*			
Penicillin	29/116 (25.0)	9/61 (14.8)	0.12
Chloramphenicol	1/115 (0.9)	3/61 (4.9)	0.09
Cotrimoxazole	9/116 (7.8)	6/60 (10.0)	0.64
Tetracycline	2/69 (2.9)	2/27 (7.4)	0.32
*H. influenzae*			
Ampicillin	27/104 (26.0)	27/58 (46.6)	0.01
Chloramphenicol	27/104 (26.0)	24/58 (41.4)	0.04
Cotrimoxazole	35/104 (33.7)	28/58 (48.3)	0.07

## Discussion

Our data demonstrate the importance of pneumococcus and Hib in the aetiology of meningitis in PNG; with the serotype distribution of pathogenic pneumococci and age of infection having implications for vaccine efficacy. Potential changes in serotype distribution (post-vaccine) and antimicrobial susceptibility dictate the need for ongoing surveillance.

Hib accounts for the vast majority of pathogenic *H. influenzae* isolated in this setting (and other settings), enabling the Hib vaccine to have a significant impact on disease. In contrast, non-PCV13 serogroups/types 2, 8, 12, 18A, 19B, 24 and 46 were amongst the most commonly isolated strains of *S. pneumoniae*, accounting for >40 % of all pneumococcal isolates in this study. The broad range of serotypes observed in this study is in keeping with our earlier study in which Lehmann et al found so-called ‘adult’ serogroups of pneumococci were more commonly isolated from CSF of children than ‘paediatric’ serotypes (i.e. 6, 14) [17].

Serotype 2, the most commonly isolated serotype in this study, has recently been described as a “newly recognised pneumococcal infection threat” [19]; however, it has been noted as a major cause of pneumococcal meningitis in PNG for the past 30 years with no obvious temporal clustering [6, 17]. Saha and colleagues noted that serotype 2 affected younger children relative to other serotypes [19]. In contrast, in our study the age distribution of serotype 2 reflected the pneumococcal isolation rate over the age groups (Table 2 and Additional file 2: Table S2). Our study provides evidence that serotype 2 presents a threat to this region. PCV13 does not include serotype 2, and upper respiratory tract carriage of other non-vaccine serotypes of pneumococcus is common in infants in this setting [20]. Thus, there may be the potential for an increase in disease due to non-vaccine serotypes post pneumococcal vaccine introduction in PNG, as was seen after the introduction of PCV7 in other settings [21].

Despite the potential for serotype replacement, the overall impact of PCVs has been positive, particularly in high income settings and with broader spectrum (PCV10 and PCV13) vaccines. It is difficult to make direct comparisons due to lack of meningitis-specific data, but in high-income settings PCVs have reduced overall IPD rates by up to 80 % as a result of the better match between serotypes in PCVs and serotypes causing disease [13]. Even with lower serotype coverage (relative to high income settings) and the potential for serotype replacement, a vaccine that offers 40–50 % coverage in a high burden setting will save lives. When consideration is given to the roll of pneumococcus in pneumonia, the case for immediate PCV rollout in high-burden settings becomes even stronger.

Our data demonstrate differences in age distribution and CFR between Hib meningitis and pneumococcal meningitis (Table 2). The CFRs for laboratory-confirmed bacterial meningitis was higher than the CFR for patients in whom no bacterial pathogen was isolated, and there was a trend towards a higher CFR in patients with pneumococcal meningitis than in those with *H. influenzae* meningitis (though not statistically significant). The CFRs observed in the current surveillance are a considerable improvement on those observed previously in the same setting, when approximately one-third of children with probable or confirmed bacterial meningitis died [17]. It is difficult to ascertain the reasons for this decrease in CFR over the two study periods; though better overall health of the population resulting in less severe disease, and/or improved management, may be contributing factors.

We observed high and increasing rates of antimicrobial resistance in Hib isolates. An increase in resistance relative to our previous study was observed, in which all Hib were susceptible to ampicillin and chloramphenicol [17]. A recent study in the lowlands of PNG found all *H. influenzae* CSF isolates tested (*n* = 14) were chloramphenicol-resistant [22]. Until recently chloramphenicol was the first-line treatment for meningitis in children in PNG: due to increasing resistance of Hib to chloramphenicol, ceftriaxone has now replaced it as the treatment of choice [23]. The observation that four Hib isolates were non-susceptible to ceftriaxone using the disk-diffusion method is of concern; however MICs were not conducted to confirm non-susceptibility (due to loss of viability of the isolates). Resistance to ceftriaxone in Hib remains uncommon in other settings [24, 25]; nonetheless, ongoing monitoring of ceftriaxone susceptibility of Hib is imperative given its current use for treatment of meningitis in PNG.

Penicillin-resistant pneumococci have long been recognised in PNG. In this study 21.5 % of isolates were penicillin resistant: a similar proportion of isolates (7/31; 22.6 %) had an MIC ≥0.125 μg/ml in the previous study conducted in this setting [17]. Thus, on the basis of current and previous findings [17, 22], there is no evidence of increasing prevalence of antimicrobial resistant pneumococci in PNG.

Tetracycline and erythromycin are not well suited for the treatment of meningitis; however, monitoring resistance to these antibiotics in pneumococcal isoaltes is of value. With limited routine diagnostic culture and sensitivity conducted in PNG, it is important to gain an insight into resistance patterns for a wide range of antimicrobial agents from relatively few clinical isolates. Moreover, baseline data on macrolide resistance in malaria endemic settings is of value as trials are conducted on malaria prophylaxis [26].

Our study provides important data leading up to the introduction of Hib and PCV vaccines. The Hib vaccine (introduced in 2008) and the PCV13 (rollout commenced in 2014) should reduce the number of cases of bacterial meningitis. The predominance of pneumococcal meningitis in the first 6 months of life highlights the need for early protection. In PNG both an accelerated 1-2-3-month PCV schedule (which ties in with PNG’s standard EPI schedule) and a schedule including a neonatal dose (0,1 and 2 months) have been shown to be safe and immunogenic [27] and should assist in protecting young children from disease caused by vaccine serotypes. Recent data from GGH show the benefit of the introduction of Hib vaccination into the national EPI program. Analysis conducted by our research team [28] reveal that the isolation rate of Hib from CSF fell significantly from 6.0 % pre-introduction (2004–7) to 0.94 % following introduction (2009–13) (*χ*2, *P* < 0.001). There was no change in the isolation rate of *S. pneumoniae* over the same period [28].

We acknowledge that there are some limitations of the study and resulting data. We isolated higher numbers of *S. aureus* than expected. Further investigation indicated that CSF collection methods were inadequate and likely to have contributed to high isolation rate of *S. aureus* in 2004–2005. Some of these isolates, and some or all of those in previous years (in which no more than 6 were isolated in any given year between 1997 and 2003) may have been the causative agent of meningitis. Of the 68 *S. aureus* isolates, 11 corresponding CSF specimens were observed to have elevated PMN counts. However, even in samples with high PMN counts we cannot discount the possibility of another undetected bacterial pathogen being the causative agent of meningitis. This cautious supposition is supported by the fact that elevated PMN counts were detected in some specimens from which no bacteria were isolated (Additional file 2: Tables S2 and Additional file 3: Table S3). Given that *S. aureus* is rarely a cause of paediatric meningitis, and is generally associated with pre-existing abnormalities of the central nervous system or recent surgery (which were not present in our patients) [29, 30], we concluded that *S. aureus* were most likely contaminants.

Our isolation rate of other contaminants (aside from *S. aureus*) was <2 %, which is consistent with other CSF culture studies (e.g. Dunbar et al. [31]). One additional limitation of our study is that we obtained data from only one site within PNG, which may not be representative of the whole country.

The benefits of ongoing multi-site surveillance of bacterial diseases in high-burden settings are well recognised; however, conducting such surveillance is costly and the level of expertise required is in short supply. At regional sites non-culture based methods could be applied. However, antigen detection assays have short-comings, and currently available culture-independent nucleic acid detection methods appear to lack the robustness and user-friendliness required for resource-poor regional settings [32]. Moreover, neither method enables antimicrobial susceptibility testing to be conducting (though resistance can be inferred through the detection of genes). Concerted efforts are required to develop expertise and methods to enable more widespread and sustainable surveillance of *S. pneumoniae* and *H. influenzae* disease and upper respiratory tract carriage, as vaccines that reduce the impact of these pathogens are introduced globally.

## Conclusions

Meningitis remains an important cause of severe childhood illness and death globally. However, vaccines are now available for two of the bacterial pathogens commonly associated with meningitis, namely *H. influenzae* type B and up to 13 serotypes of *S. pneumoniae*. Pre-vaccine surveillance data is imperative to gain an insight into the impact of the vaccines when they are introduced. Our data demonstrate the important role that non-PCV serotypes play in childhood disease in PNG. While the introduction of PCV is welcome in PNG, ongoing surveillance is imperative to monitor the role of non-vaccine serotypes in disease.

## Additional files

Additional file 1: Table S1. Overview of microscopy results for cases of suspected meningitis in children in Goroka, Papua New Guinea. In this study, unusually high rates of *S. aureus* positive CSF was detected. Microscopy results support the notion that *S. aureus* is likely a contaminant in the majority of samples from which it was isolated. (DOCX 15 kb) Additional file 2: Table S2. Prevalence of serogroups and serotypes of *S. pneumoniae* isolated from children with meningitis admitted to Goroka General Hospital. (DOCX 22 kb) Additional file 3: Table S3. Comparison of number of polymorphonucleocytes in CSF samples positive for *S. aureus* isolation versus recognised pathogens (*S. pneumoniae* and *H. influenzae*), other pathogens (non-pneumococcus, non-Hi), probable contaminants and samples from which no bacteria were isolated. There is no evidence of significance difference between PMN numbers in CSF with *S. aureus* compared to CSF with no pathogens isolated. Analysis conducted using a non-parametric independent samples median test. (DOCX 16 kb)

## Abbreviations

CFR: Case fatality rate
CLSI: Clinical and Laboratory Standards Institute
CSF: Cerebral spinal fluid
Hib: *Haemophilus influenzae* type b
GGH: Goroka General Hospital
IPD: Invasive pneumococcal disease
MIC: Minimum inhibitory concentration
PCV: Pneumococcal conjugate vaccine
PNG: Papua New Guinea
PNGIMR: Papua New Guinea Institute of Medical Research
PPV: Pneumococcal polysaccharide vaccine
