# Supplementary material for: Streptococcus pneumoniae and Haemophilus influenzae in paediatric meningitis patients at Goroka General Hospital, Papua New Guinea: serotype distribution and antimicrobial susceptibility in the pre-vaccine era
Source: BMC Infect Dis. 2015 Oct 27;15:485. doi: 10.1186/s12879-015-1197-0 (PMC4628371; doi:10.1186/s12879-015-1197-0)
Supplement: Additional file 1: Table S1. — Overview of microscopy results for cases of suspected meningitis in children in Goroka, Papua New Guinea. In this study, unusually high rates of S. aureus positive CSF was detected. Microscopy results support the notion that S. aureus is likely a contaminant in the majority of samples from which it was isolated. (DOCX 15 kb) [file 12879_2015_1197_MOESM1_ESM.docx]

Additional file 1: Table S1: Overview of microscopy results for cases of suspected meningitis in children in Goroka, Papua New Guinea. In this study, unusually high rates of *S. aureus* positive CSF was detected. Microscopy results support the notion that *S. aureus* is likely a contaminant in the majority of samples from which it was isolated.

| Bacteriology result and proportion with microscopy | PMN mean ^a^ | PMN median ^a^ | No. specimens PMN 10–100 (proportion)^b^ | No. specimens PMN > 100 (proportion)^b^ | Lymphocyte mean ^a^ | Lymphocyte median ^a^ | RBC mean ^a^ | RBC median ^a^ |
| --- | --- | --- | --- | --- | --- | --- | --- | --- |
| *S. pneumoniae* (126/180; 70%) | 1596 | 252 | 24 (19%) | 90 (71%) | 172 | 54 | 1446 | 57 |
| *H. influenzae* (104/165; 63%) | 2823 | 477 | 16 (15%) | 83 (80%) | 274 | 75 | 1934 | 84 |
| Other pathogens (22/32; 69%) | 1104 | 23 | 2 (9%) | 9 (41%) | 75 | 10 | 6831 | 78 |
| Possible pathogens ^c^ (56/76; 74%) | 143 | 0 | 6 (11%) | 7 (13%) | 19 | 0 | 875 | 0 |
| *S. aureus* ^d^ (50/68; 74%) | 159 | 0 | 4 (8%) | 7 (14%) | 21 | 0 | 923 | 0 |
| Prob contaminant (18/32; 56%) | 13 | 0 | 3 (17%) | 1 (6%) | 10 | 0 | 6327 | 270 |
| No pathogen (1249/1404; 89%) | 176 | 0 | 147 (12%) | 117 (9%) | 113 | 0 | 5490 | 1 |

^a^ Cell count values are × 10^6^/L. A value of 0 equates to <1 × 10^6^/L (below the limit of detection).

^b^ Proportion relative to samples on which microscopy was conducted.

^c^ Possible pathogens consists of *S. aureus* and other bacterial species which under specific circumstances may rarely be associated with meningitis.

^d^ *S. aureus* is a subset of ‘possible pathogens’; however is also analysed separately to clarify the role, if any, in disease.
