# Supplementary material for: Streptococcus pneumoniae and Haemophilus influenzae in paediatric meningitis patients at Goroka General Hospital, Papua New Guinea: serotype distribution and antimicrobial susceptibility in the pre-vaccine era
Source: BMC Infect Dis. 2015 Oct 27;15:485. doi: 10.1186/s12879-015-1197-0 (PMC4628371; doi:10.1186/s12879-015-1197-0)
Supplement: Additional file 2: Table S2. — Prevalence of serogroups and serotypes of S. pneumoniae isolated from children with meningitis admitted to Goroka General Hospital. (DOCX 22 kb) [file 12879_2015_1197_MOESM2_ESM.docx]

Additional file 2: Table S2: Prevalence of serogroups and serotypes of *S. pneumoniae* isolated from children with meningitis admitted to Goroka General Hospital.

| Serogroup | Number | Frequency^1^ | Serotype |
| --- | --- | --- | --- |
| 2 | 30 | 17.5 |  |
| 5 | 18 | 10.5 |  |
| 46 | 15 | 8.8 |  |
| 7 | 12 | 7.0 | 7F - 10; tnc - 2 |
| 6 | 11 | 6.4 | 6A - 1; 6B - 5; tnc - 5 |
| 4 | 10 | 5.8 |  |
| 18 | 9 | 5.3 | 18A - 3; 18C - 3; 18F - 1; tnc - 2 |
| 24 | 9 | 5.3 | 24F - 7; tnc - 2 |
| 14 | 8 | 4.7 |  |
| 12 | 7 | 4.1 | 12F - 4; tnc - 3 |
| 23 | 7 | 4.1 | 23F - 6; tnc - 1 |
| 8 | 6 | 3.5 |  |
| 19 | 6 | 3.5 | 19A - 1; 19B - 4; tnc - 1 |
| 9 | 5 | 2.9 | 9L – 1; 9V – 1; tnc - 3 |
| 45 | 5 | 2.9 |  |
| 10 | 4 | 2.3 | 10B - 1; 10F - 2; tnc - 1 |
| 1 | 2 | 1.2 |  |
| 11 | 1 | 0.6 | 11C – 1 |
| 22 | 1 | 0.6 | 22A - 1 |
| 27 | 1 | 0.6 |  |
| 28 | 1 | 0.6 |  |
| 29 | 1 | 0.6 |  |
| 32 | 1 | 0.6 |  |
| 33 | 1 | 0.6 | tnc |

^1^ Frequency: Proportion (%) of all serogrouped isolates (n=171). One additional isolate was non-serotypable. tnc: typing not conducted.
