# Supplementary material for: Streptococcus pneumoniae and Haemophilus influenzae in paediatric meningitis patients at Goroka General Hospital, Papua New Guinea: serotype distribution and antimicrobial susceptibility in the pre-vaccine era
Source: BMC Infect Dis. 2015 Oct 27;15:485. doi: 10.1186/s12879-015-1197-0 (PMC4628371; doi:10.1186/s12879-015-1197-0)
Supplement: Additional file 3: Table S3. — Comparison of number of polymorphonucleocytes in CSF samples positive for S. aureus isolation versus recognised pathogens (S. pneumoniae and H. influenzae), other pathogens (non-pneumococcus, non-Hi), probable contaminants and samples from which no bacteria were isolated. There is no evidence of significance difference between PMN numbers in CSF with S. aureus compared to CSF with no pathogens isolated. Analysis conducted using a non-parametric independent samples median test. (DOCX 16 kb) [file 12879_2015_1197_MOESM3_ESM.docx]

Additional file 3: Comparison of number of polymorphonucleocytes in CSF samples positive for *S. aureus* isolation versus recognised pathogens (*S. pneumoniaae* and *H. influenzae*), other pathogens (non-pnc, non-Hi), probable contaminants and samples from which no bacteria were isolated. There is no evidence of significance difference between PMN numbers in CSF with *S. aureus* compared to CSF with no pathogens isolated. Analysis conducted using a non-parametric independent samples median test.

| Bacteriology result | P value |
| --- | --- |
| *S. pneumoniae* | 0.000 |
| *H. influenzae* | 0.000 |
| Other pathogens | 0.000 |
| Probable contaminants | 0.000 |
| No pathogen | 0.239 |
